# Evaluation of the relationship between visual examination and loupes usage in caries detection and dentists’ fatigue levels: an in-vitro study

**DOI:** 10.1186/s12903-026-08707-7

**Published:** 2026-05-28

**Authors:** Deniz Çetin, Aslı Silkü Oflioğlu, Burçin Aydemir Akkoçan, Hülya Erten Can

**Affiliations:** Faculty of Dentistry, Department of Restorative Dentistry, Dokuz Eylül University, Izmir, Türkiye

**Keywords:** Caries detection, Fatigue, Histology, Loupe, Visual examination

## Abstract

**Background:**

The aim of this study is to evaluate the effect of visual and general fatigue on intra- and inter-observer reliability in caries assessment using the ICDAS II system, and to investigate the role of magnification in maintaining examiner consistency under different fatigue conditions.

**Methods:**

Extracted teeth without decay or with early enamel lesions were embedded in silicone impression material. Dentists’ visual and physical fatigue levels were assessed twice daily—before morning practice and after afternoon sessions—using the Visual Fatigue Questionnaire and the Chalder Fatigue Scale. Three restorative dentistry research assistants with similar clinical experience examined the teeth using visual inspection and loupe magnification based on the ICDAS II criteria. Histological evaluation was performed under 10× magnification using the ERK (Ekstrand, Ricketts and Kidd) Histological Scoring System. ICDAS II scores were compared with histological scores. Histological evaluation was used as a reference framework rather than a direct measure of diagnostic accuracy. Examiners repeated measurements one week apart to calculate Intraclass Correlation Coefficient (ICC) values for intra-observer reliability. Weighted Cohen’s Kappa analysis assessed inter-observer agreement, the relationship between diagnostic methods, and the effect of fatigue.

**Results:**

Loupe use significantly increased ICC values for all observers (≈ 0.22; *p* < 0.001). In visual examination, ICC values ranged from 0.39 to 0.86, whereas loupe-assisted evaluations yielded ICC values of 0.81–0.95, indicating substantially improved intra-observer reliability. Weighted Cohen’s Kappa revealed significant morning–afternoon intra-observer agreement (*p* < 0.001), with Kappa values of 0.62–0.84 in the morning and 0.54–0.73 in the afternoon. Loupe-assisted examinations consistently showed higher diagnostic agreement, while decreased examiner consistency in the afternoon was associated with visual fatigue and reduced attention. Inter-observer agreement was also significant and high for both methods (*p* < 0.001). Morning Kappa values were 0.66 for visual examination and 0.69 for loupe-assisted examination; afternoon values were 0.59 and 0.61, respectively. Overall, loupe use was associated with improved examiner consistency and agreement and afternoon declines reflected the impact of fatigue.

**Conclusion:**

Within the limitations of this study, the use of loupes significantly improved intra-observer reliability and observer consistency in caries detection. Additionally, fatigue accumulated during the day negatively affected examiner reliability, as reflected by decreased agreement levels in afternoon assessments. These findings emphasize the role of visual support tools and operator-related factors in maintaining diagnostic consistency.

## Background

Clinical examination, caries detection, shade selection, and all minimally invasive restorative approaches are directly related to the dentist’s visual performance. Repetitive procedures over long hours during the day, exposure to intense light sources, use of digital screens, and intense clinical hours are identified as key factors accelerating the development of physical, mental and eye fatigue in dentists [1, 2]. Eye fatigue causes a decrease in focusing power, a drop in contrast sensitivity, weakened colour discrimination, blurred vision, excessive sensitivity to light, and loss of attention [3].

Caries detection, one of the cornerstones of restorative dental treatment, is largely dependent on the dentist’s visual assessment. The diagnostic accuracy of visual examination methods is directly related to the dentist’s level of attention and visual capacity. The literature indicates that decreased visual performance leads to the omission of initial enamel lesions, the overlooking of low-contrast opaque areas, and an increase in classification errors [4, 5]. Fatigue-related decrease in attention reduces decision-making speed and can increase both false negative and false positive diagnosis rates [6]. Studies have shown that diagnostic accuracy decreases in dental examinations performed later in the day, and the rate of overlooking initial caries lesions in particular increases [7]. While previous studies have primarily focused on diagnostic accuracy, the present study specifically aims to evaluate examiner reliability and consistency under fatigue conditions.

Another critical area where the effects of eye fatigue become apparent is the colour selection process. Achieving successful results in aesthetic restorations depends on accurately determining the natural tooth colour, which requires high precision in distinguishing parameters such as value, chroma, hue, and translucency. However, visual fatigue makes it particularly difficult to perceive colour differences, increasing the likelihood of incorrect colour selection due to changes in retinal adaptation [8]. Studies report that eye fatigue leads to a loss of selectivity in A–B group shades, inconsistency in opacity assessments, and the risk of colour mismatch in restorations [9, 10]. This situation leads to both the need for repeat treatment and reduced patient satisfaction.

The proliferation of digital photography, CAD/CAM systems, and high-intensity dental lighting in modern dental practice has further increased the visual load on dentists. Prolonged exposure to bright screens reduces the blink rate, triggering dry eyes and accelerating fatigue [11]. When these factors combine, they can result in a decline in visual performance that may affect the quality of restorative clinical decisions.

In this context, it is important to scientifically evaluate the effect of practitioner fatigue on diagnostic processes in restorative dental treatment. Identifying the effects of fatigue on caries detection and colour determination accuracy will provide critical data for establishing safer clinical working practices, determining ideal working times, and developing ergonomic lighting and digital workflows. This study evaluated the effect of eye fatigue and general mental/physical fatigue in dentists on caries detection. The aim of this study is to evaluate the effect of visual and general fatigue on intra- and inter-observer reliability in caries assessment using the ICDAS II system, and to investigate the role of magnification in maintaining examiner consistency under different fatigue conditions.

## Methods

**Fig. 1:**
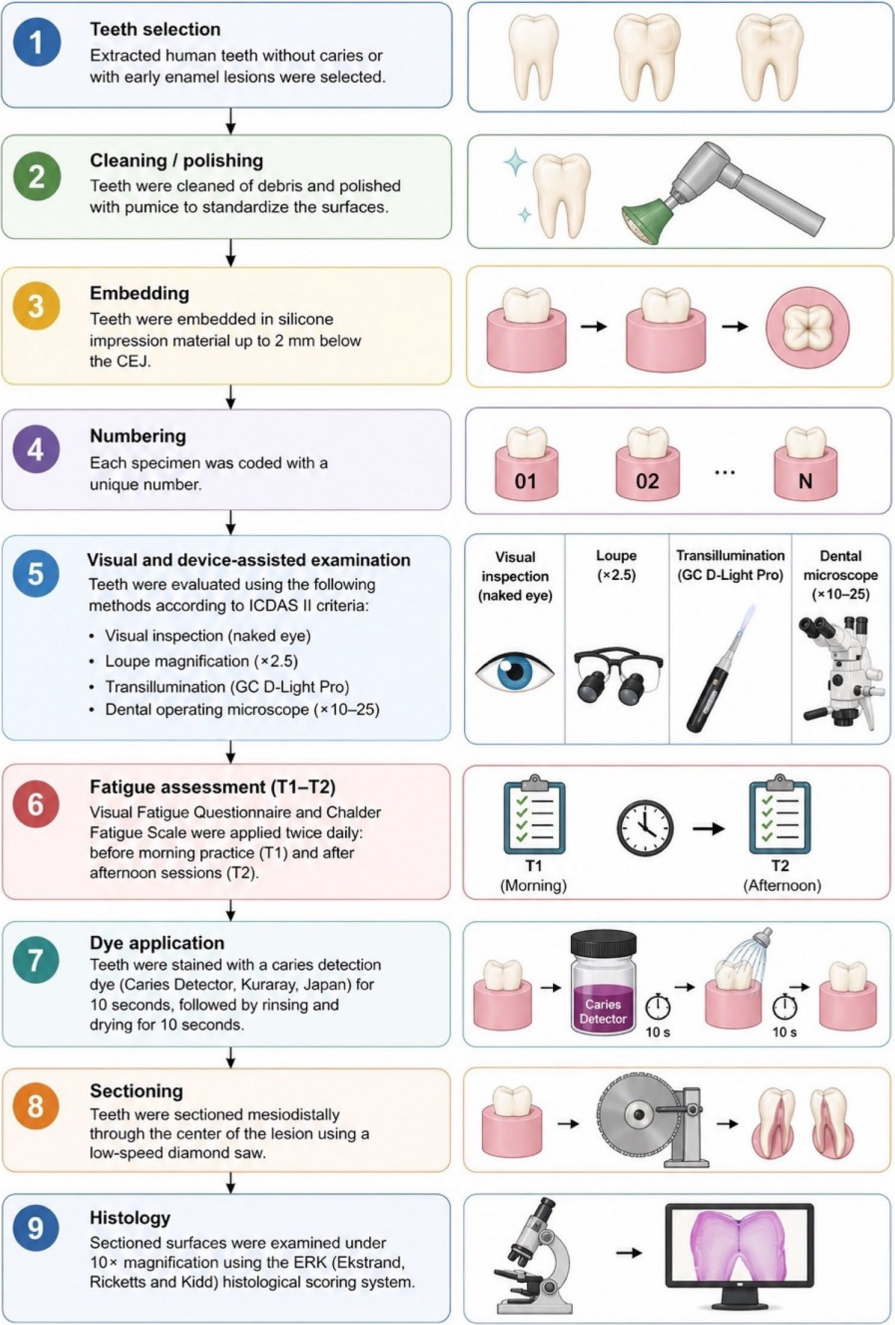
Schematic representation of the study workflow

A total of 128 extracted posterior teeth with non-carious or early-stage occlusal caries were collected. The teeth were stored in distilled water until they were used in the study. Subsequently, their occlusal surfaces were polished with prophylaxis paste. The prepared teeth were embedded in silicone impression material, and each tooth was assigned a number (Fig. 1).

This study received ethical approval from Research Ethics Committee (REC) of Faculty of Medicine, Dokuz Eylül University (Approval Number: 2026/03–12), in compliance with the Declaration of Helsinki and its subsequent amendments. Written informed consent was obtained from all participants included in the study.

Cavities were examined by three restorative dentistry research assistants with equal clinical experience, using each of the two diagnostic methods (visual examination and loupe) according to the ICDAS II classification system.

**Fig. 2:**
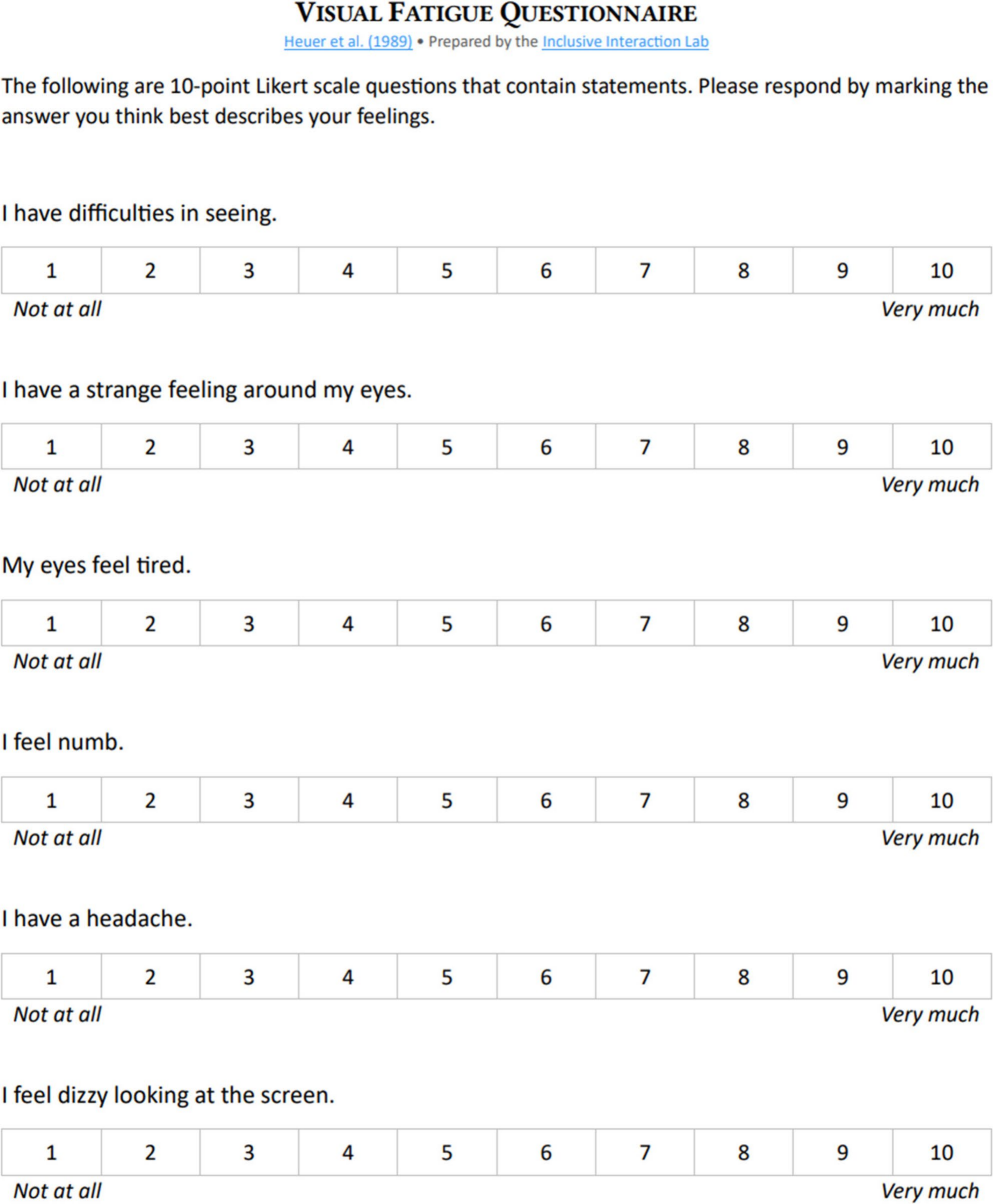
Visual fatigue questionnaire

Prior to the examinations, the ‘‘Visual Fatigue Questionnaire’’ and the ‘‘Chalder Fatigue Scale’’ were administered to the dentists twice: once before they began treating patients in the morning and once after completing treatment for all patients in the afternoon, in order to measure the dentists’ visual and physical fatigue during the day (Fig. 2).

**Fig. 3:**
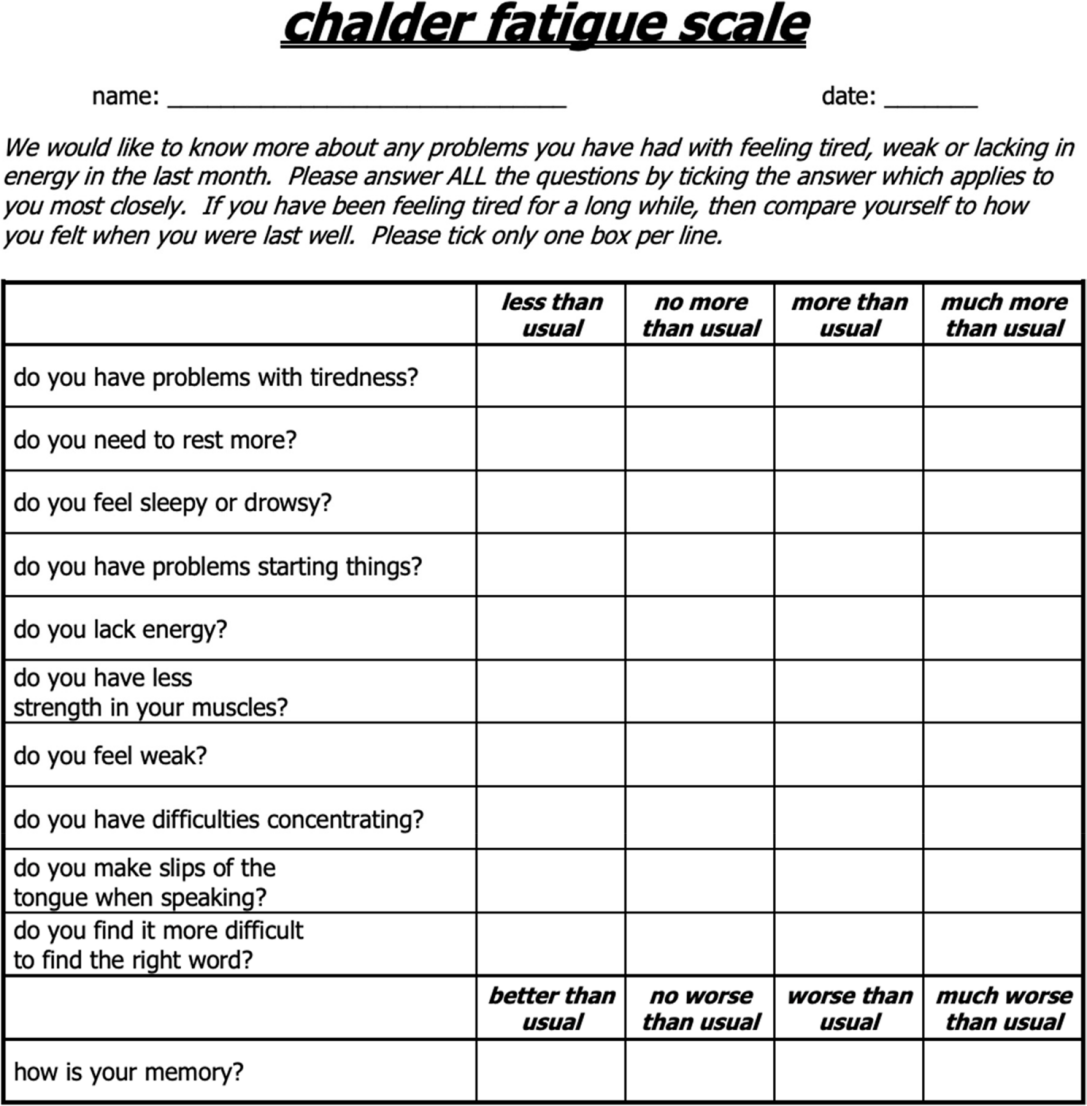
Chalder fatigue scale

The Visual Fatigue Questionnaire was developed by Heuer and colleagues in 1989 and introduced into the literature. The purpose of the questionnaire is to subjectively measure the symptoms of visual fatigue. This questionnaire consists of 6 questions. A Likert-type scale, which can be scaled from 1 to 10 for each question, is used. This questionnaire is generally used in the literature to assess the effects of visual fatigue symptoms on diagnostic accuracy (Fig. 3).

The Chalder Fatigue Scale was first introduced to the literature by Chalder and colleagues in 1993 and has been refined over the years. It consists of 11 questions in total. The first 7 questions aim to subjectively assess participants’ physical fatigue symptoms, while the remaining 4 questions assess mental fatigue symptoms. Each question is scored using a 4-point Likert scale (“better than usual” = 0, “no different from usual” = 1, “worse than usual” = 2, “much worse than usual” = 3). The total score ranges from 0 to 33, with higher scores indicating more severe fatigue.

Visual examination was performed after drying the occlusal surfaces of the teeth with air, using a probe under reflected light in a manner that would not damage the teeth. Subsequently, examinations were performed using a x2.5 magnifying ZEISS EyeMag Smart (Carl Zeiss Meditec AG, Germany) dental loupe.

**Fig. 4:**
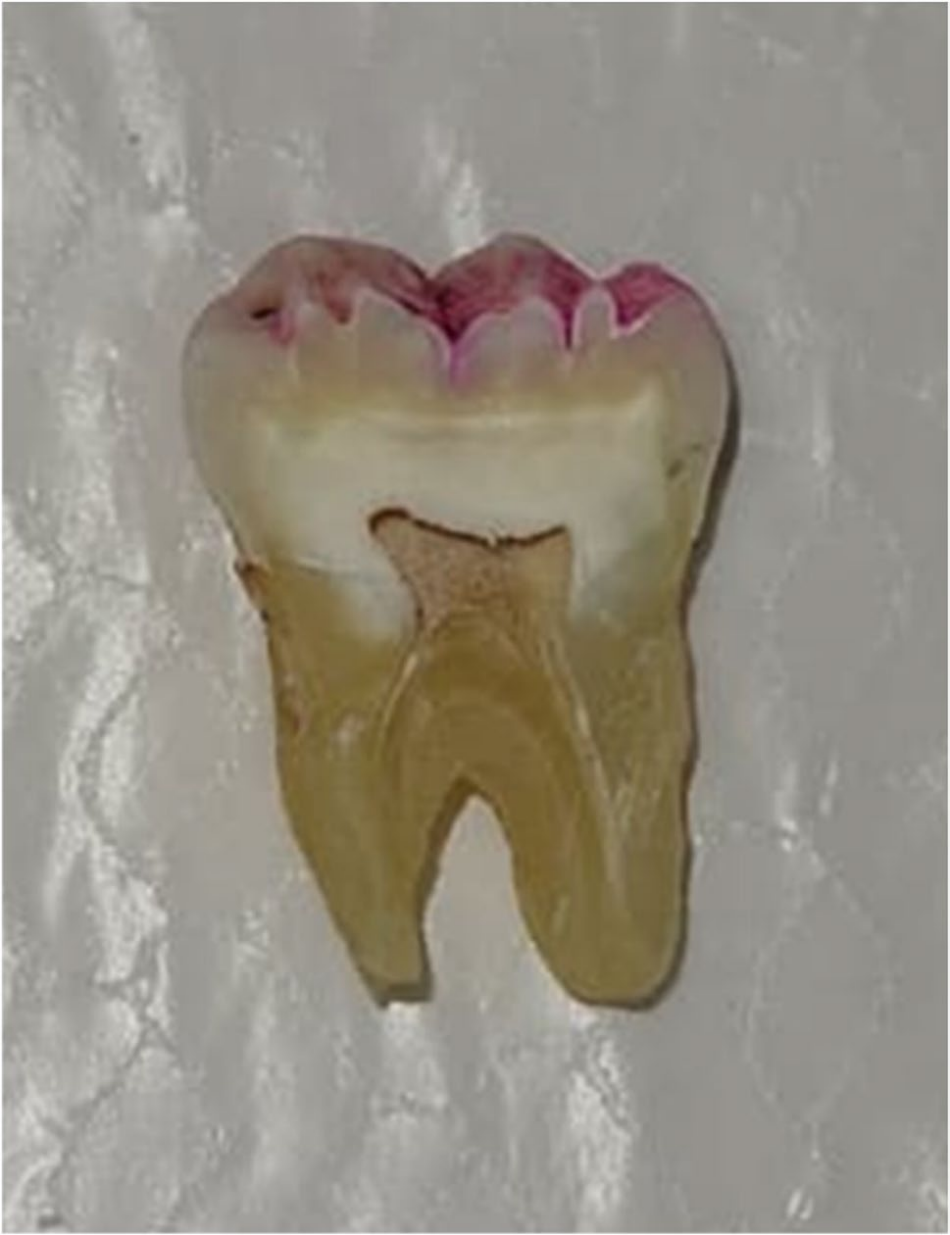
Tooth sectioned in half after staining with 1% acid red

Once all examinations were completed, the teeth were stained with a caries detection dye (Caries Detector, Kuraray, Japan) containing 1% acid red (Fig. 4). The teeth were left in the dye for 10 s, then rinsed and dried for 10 s. The teeth were then sectioned into half-cuts under water using a diamond separator, parallel to the long axis of the tooth, along the fissure pattern in the mesiodistal direction, passing through the exact centre of the occlusal fossa. Subsequently, the teeth were histologically examined under a stereomicroscope (Olympus, Japan) at 10× magnification.

Cavities were assessed based on the ERK (Ekstrand, Ricketts and Kidd) Histological Scoring System. ICDAS II scores were compared with histological scores (Table 1). Histological evaluation was used as a reference framework rather than a direct measure of diagnostic accuracy.

**Table 1:** Comparison of ICDAS II and ERK scores

ICDAS II Score (Clinical)	Clinical Description	ERK Score (Histology)	Histological Description
0	Sound tooth surface (no signs of lesion).	0	No demineralization.
1–2	Initial lesions (visually detectable enamel demineralization).	1	Demineralization confined to the enamel.
3	Localized enamel breakdown (small cavity, dentin not exposed).	2	Lesion has reached the DEJ with limited progression into dentin.
4	Dark shadow (indicates dentin caries, surface typically intact).	3	Lesion has progressed to a moderate depth within the dentin.
5–6	Cavitated lesions: extensive dentin cavity (5: medium depth; 6: deep cavity reaching the pulp).	4	Lesion has progressed deeply into the dentin toward the pulp.

### Statistical analysis

To test consistency, each observer repeated the examinations twice, with a one-week interval between sessions, and the Intraclass Correlation Coefficient (ICC) value was calculated. Kappa Analyses were applied to assess inter-observer agreement, the relationship between methods, and the observers’ morning and afternoon fatigue scores. In the evaluation of the Chalder Fatigue Scale, the mean and standard deviation values and the results of the Paired t-Test were calculated.

## Results

The average values of the *Visual Fatigue Questionnaire* measurements taken in the morning and afternoon are given in Table 2. This scale was administered to observers designated by the letters A, B, and C before the examinations in the morning and afternoon. The average morning (T1) visual fatigue scores were found to be in the range of 10–15. In the scale applied in the afternoon (T2), the average visual fatigue scores were in the range of 21–29. Considering the average values of all observers, it was determined that the values measured in the afternoon showed an increase compared to those measured in the morning. The average increase score was calculated as 13. These values indicate that the visually-measured fatigue levels of physicians subjectively increased significantly at the end of a routine clinical working day (Table 2).

**Table 2:** Average values of the ‘*Visual Fatigue Questionnaire’* in the morning and afternoon measurements

Observer	Morning (T1)	Afternoon (T2)	Increase (T2–T1)
A	12	29	+ 17
B	15	26	+ 11
C	10	21	+ 11
Average	12.3	25.3	+ 13

The results of the survey conducted using the *Chalder Fatigue Scale* are presented in Table 3. This scale was administered to observers designated by the letters A, B, and C before examinations in the morning and afternoon. The average morning (T1) fatigue scores were found to be in the range of 9–11. This result indicates a ‘low level of fatigue’. In the scale applied in the afternoon (T2), the average score was in the range of 18–21. This result indicates a value of ‘moderate to high level of fatigue’. Considering the average values of all observers, it was determined that the values measured in the afternoon showed an increase compared to those measured in the morning. The average increase in score was calculated as 9.67 ± 2.08. When evaluated with a paired t-test, this increase was found to be statistically significant (*p* ≈ 0.015; *p* < 0.05). These values indicate that physicians’ subjectively measured fatigue levels increase significantly at the end of a routine clinical working day (Table 3).

**Table 3:** Mean values of the Chalder Fatigue Scale in morning and afternoon measurements

Observer	Morning (T1)	Afternoon (T2)	Δ (T2 − T1)
A	9	21	+ 12
B	10	18	+ 8
C	11	20	+ 9
Average ± SD	10.0 ± 1.0	19.67 ± 1.53	9.67 ± 2.08

*SD* Standard deviation, Δ difference

The Intraclass Correlation Coefficient (ICC) values for measurements repeated by observers at one-week intervals to test consistency are shown in Table 4.

**Table 4:** Intraclass Correlation Coefficient (ICC) value of measurements repeated by observers at one-week intervals to test consistency

Observer	Period	Examination Method	ICC (κ)	Agreement Level (Cicchetti, 1994)
A	T1	Visual	0.57	Moderate
A	T1	Loupe	0.94	Excellent
A	T2	Visual	0.39	Poor
A	T2	Loupe	0.81	Excellent
B	T1	Visual	0.86	Excellent
B	T1	Loupe	0.84	Excellent
B	T2	Visual	0.69	Good
B	T2	Loupe	0.87	Excellent
C	T1	Visual	0.80	Excellent
C	T1	Loupe	0.95	Excellent
C	T2	Visual	0.73	Good
C	T2	Loupe	0.92	Excellent

*T1* Morning, *T2 *Afternoon; *p* < 0.001

In all observers, the use of a loupe during examination resulted in a significant increase in ICC values (≈ 0.22; *p* < 0.001). While ICC values ranged from 0.39 to 0.86 in visual examinations, they increased to the range of 0.81–0.95 in examinations performed using a loupe. These results demonstrated that the use of a loupe significantly increased intra-observer reliability and diagnostic consistency in caries detection.

The intra-observer reliability values for the morning and afternoon measurements are shown in Table 5.

**Table 5:** Intra-observer consistency in measurements taken in the morning and afternoon

Observer	Period	Weighted Kappa (κ)	Agreement Level (Landis & Koch, 1977)
A	T1	0.62	Good
A	T2	0.54	Moderate
B	T1	0.84	Excellent
B	T2	0.64	Good
C	T1	0.79	Good
C	T2	0.73	Good

*T1* Morning, *T2* Afternoon; *p* < 0.001

Weighted Cohen’s Kappa analysis revealed that intra-observer agreement in the morning and afternoon examinations was statistically significant for all observers (*p* < 0.001). Kappa values ranged from 0.62 to 0.84 in the morning and from 0.54 to 0.73 in the afternoon. Higher diagnostic consistency was achieved in loupe-assisted examinations, and it was thought that the afternoon decline could be due to visual fatigue and decreased attention.

These findings showed that, in general, the use of loupes increased diagnostic reliability, but that fatigue or attention differences over time could lead to small changes in Kappa values.

The inter-observer agreement values for the measurements taken in the morning and afternoon are shown in Table 6.

**Table 6:** Inter-observer agreement in measurements taken in the morning and afternoon

Period	Examination Method	Mean Kappa (κ)	Agreement Level (Landis & Koch, 1977)
T1	Visual	0.66	Good
T1	Loupe	0.69	Good
T2	Visual	0.59	Moderate
T2	Loupe	0.61	Good

*T1* Morning, *T2* Afternoon; *p* < 0.001

In the Weighted Cohen’s Kappa analysis measuring inter-observer agreement, a significant and high level of consistency was observed in both methods (*p* < 0.001). In the morning assessments, the Kappa values were 0.66 for visual examination and 0.69 for loupe-assisted examination; in the afternoon, they were 0.59 and 0.61, respectively. Overall, the use of loupes increased diagnostic reliability, and the afternoon decline was associated with visual fatigue and decreased attention.

## Discussion

A review of the literature reveals that studies measuring the visual and physical fatigue of physicians during the day have generally been conducted in the field of radiology.

In a study conducted by Krupinski and colleagues in 2010, 20 radiologists interpreted 60 bone fracture cases radiographically at the beginning and end of a routine working day. Visual fatigue and concentration measurements were taken before and after the interpretations. The findings showed that diagnostic accuracy decreased and visual concentration errors increased significantly at the end of a full working day. The results demonstrate that visual fatigue negatively affects radiological interpretation performance [12].

Krupinski and colleagues’ 2012 study aimed to test whether the fatigue-related decline in diagnostic performance in the interpretation of static bone images also applies to dynamic imaging. CT images were evaluated for this purpose. Similar to their 2010 study, the results showed that long working days also had a negative effect on radiological diagnosis in dynamic CT images [13]. Recent studies have also highlighted the role of visual ergonomics and operator fatigue in diagnostic performance, emphasizing that prolonged clinical workload and visual strain may significantly impair diagnostic accuracy [14, 15].

There are studies evaluating the effectiveness of magnification in caries detection and comparing it with the effectiveness of visual examination.

Studies by Gupta et al. [16] and Goel et al. [17] showed that the effectiveness of magnification in caries detection was higher than visual examination in detecting initial caries lesions. Gupta et al. reported that magnification was as effective as DIAGNOdent in detecting initial caries lesions on flat surfaces; Goel et al. reported that examination using a loupe on occlusal surfaces increased sensitivity to 97% [16, 17]. However, these findings mainly address diagnostic sensitivity rather than examiner reliability, which represents a distinct methodological outcome.

Ari et al. [18] also noted that magnification and good lighting improve dentists’ performance in detecting caries, particularly facilitating the detection of early caries lesions. These findings demonstrate that magnification may provide visual and ergonomic support during caries assessment; however, it should not be interpreted as an essential tool that improves diagnostic accuracy in routine examinations [18]. In contrast, the present study does not aim to evaluate diagnostic accuracy, but rather focuses on examiner reliability and consistency under fatigue conditions.

The findings of this study should be interpreted within the framework of existing cariology consensus, which supports the adequacy of visual caries assessment using the ICDAS system. The present study does not aim to challenge or replace this approach.

However, the literature presents conflicting findings regarding the effectiveness of magnification in caries detection. While several studies report increased diagnostic sensitivity, others suggest that higher magnification levels may lead to overestimation of lesion severity and increased false-positive rates. These discrepancies may be attributed to differences in magnification levels, examiner experience, lighting conditions, and lesion characteristics.

In addition to the benefits of magnification in caries diagnosis, there are also some limitations. For example, examinations performed with magnification may cause caries lesions to be perceived as more serious than they are, leading to premature or excessive treatment.

Neuhaus et al., in a study evaluating the occlusal surfaces of extracted teeth without magnification, using a Galilean-type loupe with 2.5x magnification, a Keplerian-type loupe with 4.5x magnification, and a surgical microscope with 10x magnification, reported that ICDAS assessments achieved optimal accuracy within the 1–2x magnification range [19].

These results indicate that the effect of magnification depends on the magnification level, surface type, lighting conditions, and the observer’s experience; they also highlight the need to consider the risks of false-positive diagnoses and unnecessary restorative interventions.

Unlike many previous studies that primarily focus on diagnostic accuracy, the present study specifically evaluated intra- and inter-observer reliability under varying fatigue conditions. The findings suggest that magnification may function as an ergonomic support tool that helps maintain examiner consistency, rather than directly improving diagnostic accuracy. This distinction is critical, as reliability reflects the reproducibility of assessments, whereas accuracy relates to correctness relative to a reference standard.

This study has several limitations. First, it was conducted under in vitro conditions, which may not fully reflect clinical practice. Second, the number of observers was limited. Third, fatigue was evaluated using subjective scales rather than objective physiological parameters. Future studies should include clinical settings, larger samples, and objective fatigue biomarkers.

## Conclusion

Within the limitations of this study, the use of loupes significantly improved intra-observer reliability and observer consistency in caries detection. Additionally, fatigue accumulated during the day negatively affected examiner reliability, as reflected by decreased agreement levels in afternoon assessments. These findings emphasize the role of visual support tools and operator-related factors in maintaining diagnostic consistency.

## Data Availability

The datasets used and/or analyzed during the current study available from the corresponding author on reasonable request.

## Abbreviations

CAD/CAM: Computer-Aided Design / Computer-Aided Manufacturing
ICC: Intraclass Correlation Coefficient
ICDAS: International Caries Detection and Assessment System
ERK: Ekstrand, Ricketts and Kidd
